# Testing for Differences in Consumer‐Based Nutrient Cycling Between Male and Female Wolf Spiders ( *Hogna carolinensis* )

**DOI:** 10.1002/ece3.72640

**Published:** 2025-12-05

**Authors:** Colton Herzog, Jamie T. Reeves, Shawn M. Wilder

**Affiliations:** ^1^ Department of Biology Oklahoma State University Stillwater Oklahoma USA

**Keywords:** ecological stoichiometry, intraspecific differences, ionomics, nutrient cycling, trace elements, wolf spiders

## Abstract

Assessing how elements are transferred through ecosystems provides key insights into nutrient cycling, food web interactions, and ecosystem functioning. However, studies investigating the effects of intraspecific variation on organismal elemental composition remain limited, especially for trace elements. This study investigated sex‐based differences in the elemental content of whole body and excreta of *Hogna carolinensis* (Carolina wolf spiders). We hypothesized that males and females would differ in whole‐body and excreta elemental composition due to divergent life history strategies. Our findings partially supported our hypothesis. Male spiders had significantly higher whole‐body concentrations of Fe, Ni, and S, whereas females had significantly more Sr. In excreta, males excreted significantly greater concentrations of Ca, Mn, Si, and Zn, while females excreted significantly higher concentrations of K and P. Principal component analysis revealed distinct elemental profiles between excreta and whole‐body samples, with both sexes exhibiting higher concentrations of Ba, Ca, Fe, K, N, Na, P, S, and Si in excreta, and lower concentrations of Cu, Li, and Ni than in their whole body. These results suggest that sex‐specific excretion patterns may influence micronutrient cycling and ecosystem function, particularly regarding the deposition of nutrients such as Ca, K, Mn, P, Si, and Zn. Given the potential ecological implications of sex‐based nutrient fluxes, future research should further examine how intraspecific variation and sexual dimorphism shape stoichiometric phenotypes and trophic interactions.

## Graphical abstract

We tested whether male and female wolf spiders ( *Hogna carolinensis* ) differ in how they regulate and excrete micronutrients. Our ecological experiment revealed significant differences in whole‐body and excreta elemental composition between sexes, providing new insight into how predator physiology shapes predator‐mediated nutrient cycles.

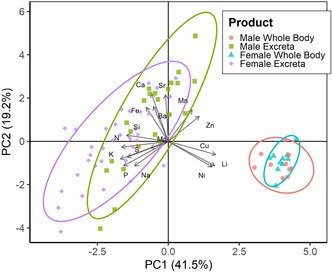

## 1 Introduction

Biological stoichiometry uses chemical ratios to describe and explain how nutrients flow between organisms and ecosystems, especially using the most biologically abundant macronutrient elements (e.g., C, N, and P) (Elser and Urabe 1999; Fagan et al. 2002; Sterner and Elser 2002; Elser et al. 2016). Recent attention has expanded towards exploring organismal stoichiometry with a focus on micronutrient elements (i.e., B, Ca Co, Cr, Cu, Fe, I, K, Li, Mg, Mn, Mo, Na, Ni, S, Si, and Zn) (Filipiak 2016; Filipiak and Weiner 2017; Filipiak 2018; Welti et al. 2019; Kaspari 2021; Welti and Kaspari 2021; Filipiak et al. 2021; Filipiak and Filipiak 2022; Herzog et al. 2023). Despite their low concentrations in life's biomass (< 1%), some of these micronutrients are critical in trophic interactions due to their necessity for ion regulation or as cofactors of vital enzymes (Welti et al. 2019; Kaspari 2021; Welti and Kaspari 2021). Furthermore, there is a growing body of literature demonstrating that consumer‐driven cycling of these micronutrients can significantly impact the function of both higher and lower trophic levels (Schmitz et al. 2010; Schmitz et al. 2014; Wilder and Schneider 2017; Schmitz et al. 2018; Welti et al. 2019; Zhou and Declerck 2019; Welti and Kaspari 2021; Kaspari 2021).

Building on this framework, recent research has explored how the micronutrient composition of organisms influences broader ecological processes (Filipiak 2016; Filipiak and Weiner 2017; Filipiak 2018; Zhou and Declerck 2019; Kaspari 2021; Welti and Kaspari 2021; Filipiak and Filipiak 2022; Herzog et al. 2023; Zhang et al. 2023). More specifically, the biology and stoichiometry of predators, especially those that serve as prey to other organisms (e.g., spiders, beetles, fish, etc.), can cascade through food webs, affecting both macro‐ and micronutrient availability across trophic levels (Sterner and Elser 2002; Filipiak 2016; Filipiak and Weiner 2017; González et al. 2017; Filipiak 2018; Welti et al. 2019; Kaspari 2021; Welti and Kaspari 2021; Filipiak et al. 2021; Filipiak and Filipiak 2022). For example, linyphiid spiders emerge early in arctic habitats, depositing ingested nutrients from prey and organic matter (i.e., pollen) into the environment (Hodkinson et al. 2001). Furthermore, neotropical jumping spiders deposit nutrients to bromeliad plants they inhabit via excreta and discarded prey remains, increasing both nutrient availability and plant fitness (Romero et al. 2006). Thus, predator excreta and unconsumed parts of prey contribute to nutrient availability for lower trophic levels (Hilderbrand et al. 1999; Barret et al. 2005; Bump et al. 2009; Nyffeler and Birkhofer 2017; Trubl and Johnson 2019; Herzog et al. 2023). Moreover, as prey items, the stoichiometry of animals influences nutrient availability to upper trophic levels (González et al. 2017; Goos et al. 2017; Ludwig et al. 2018; Sobcyzk et al. 2020; Filipiak et al. 2021). Therefore, gaining a comprehensive understanding of a predator's biology, stoichiometric phenotype, and the subsequent effects of predator–prey relationships on ecosystem function is vital for understanding the flow of nutrients through food webs.

In particular, the overall flow of elements across trophic levels may relate to stoichiometric differences not only between prey and predators, but also within species themselves. For instance, recent research highlights intraspecific variation in stoichiometric phenotypes within species, stemming from sexual dimorphism (Goos et al. 2017; Sobcyzk et al. 2020; Filipiak et al. 2021). Differences in nutrient requirements, physiology and life history strategies between sexes may contribute to variations in observed stoichiometric phenotypes (Morehouse et al. 2010; Leal et al. 2017). Body size differences between males and females may also lead to the consumption of different prey types, which could further contribute to sex‐specific elemental profiles. Additionally, stoichiometric differences between juveniles and adults have been documented in other arthropods, suggesting that developmental stage may also influence elemental composition (Babczyńska et al. 2011; Filipiak et al. 2021). Consequently, significant demographic changes in a population (e.g., shifts in sex ratios) could impact nutrients cycled in an ecosystem (Goos et al. 2017; Sobcyzk et al. 2020; Filipiak et al. 2021). However, a significant knowledge gap exists in our understanding of how intraspecific variation affects stoichiometric phenotypes and, subsequently, may influence nutrient feedback cycles. Understanding whole‐body elemental content is key to addressing this gap, as it reflects the internal nutrient pools retained by individuals, integrates physiological demands, and indicates what nutrients are ingested by a predator when a spider is consumed versus those lost through excretion.

**FIGURE 1:**
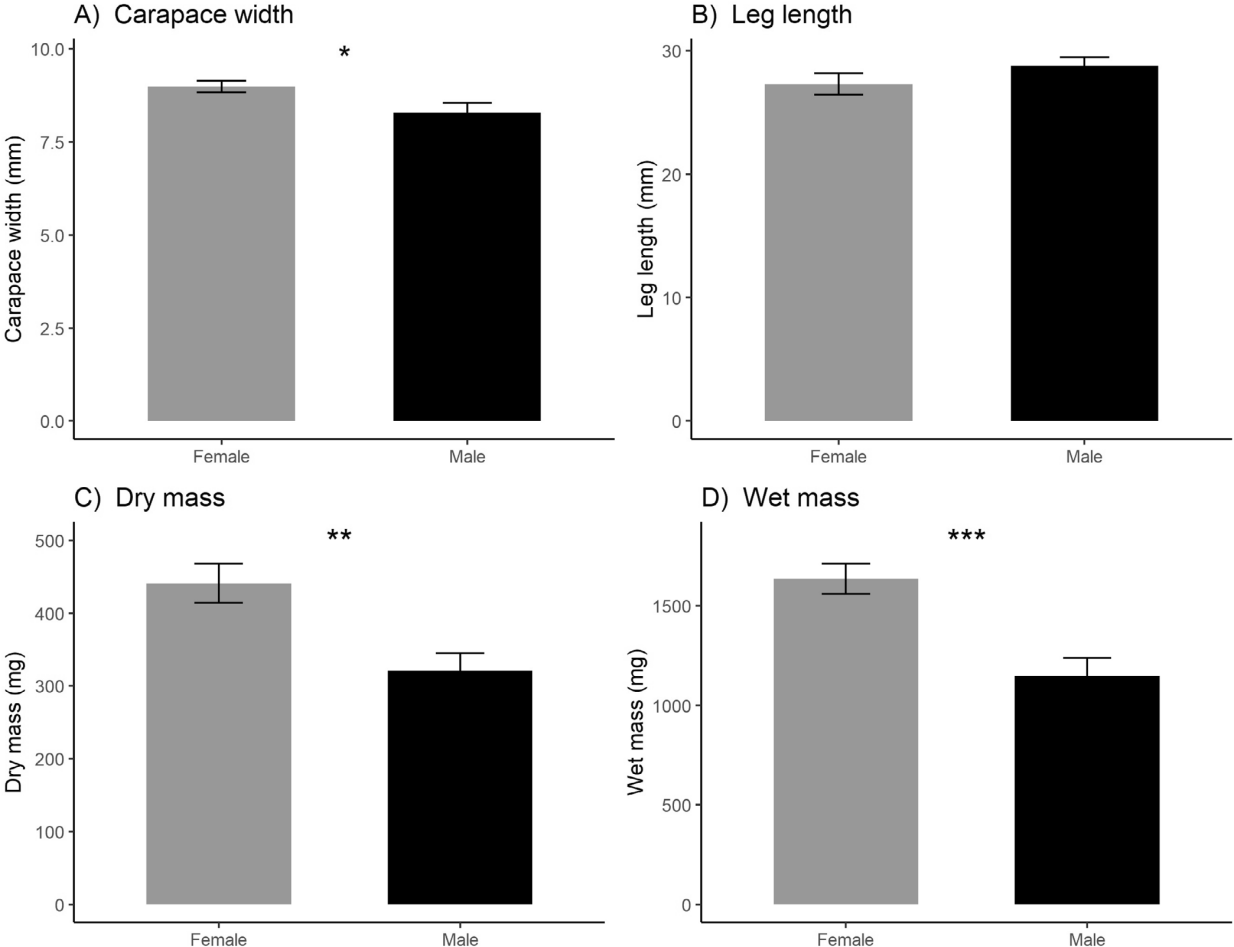
Mean (± SE) trait values for male and female wolf spiders. Significant *p*‐values are indicated with asterisks (*p < 0.05, **p < 0.01, ***p < 0.001).

Previous work on adult female Carolina wolf spiders ( *Hogna carolinensis* ) showed that spider excretion remained similar in elemental content even when they fed on different species of prey (Herzog et al. 2023). Yet, it remains unknown if both whole body and excreta nutrient content differ among male and female *H. carolinensis* . The life history strategies of *H. carolinensis* diverge significantly between the sexes. Females, with an estimated lifespan of 2–3 years, inhabit burrows until reproduction and are larger in mass (Figure 1) (Punzo 2003). Additionally, females accumulate a large amount of nutrients in their bodies to produce clutches of eggs. However, males have shorter lifespans (i.e., ~1 year; Punzo 2003), are smaller in mass, and primarily use nutrients to fuel their search for adult females (Foellmer and Moya‐Larano 2007; Cordellier et al. 2020). Hence, there could be intraspecific differences in whole‐body elemental content between the sexes that could influence their assimilation versus excretion of a variety of elements.

We hypothesized that male and female spiders would differ in their whole‐body and excreta elemental content (Ba, Ca, Cu, Fe, K, Li, Mg, Mn, N, Na, Ni, P, S, Si, Sr., and Zn). However, it is difficult to make general a priori predictions about specific trace elemental differences between whole body male and female *H. carolinensis* and their excreta due to both the number of elements that will be analyzed and the current knowledge gap in the literature regarding elemental requirements for spiders. Adult males do not experience significant growth or produce biological products, aside from small quantities of sperm. As a result, they may not require high concentrations of the micronutrients required for tissue development, instead focusing more on energy to fuel mate searching. In contrast, adult females may need higher concentrations of some micronutrient elements to support egg production and sustain longer lifespans compared to their male counterparts.

## 2 Methods

### 2.1 Study Species for Spider Excreta Collection

We collected adult male (*n* = 26) and adult female (*n* = 18) Carolina wolf spiders ( *Hogna carolinensis* ) from fields in Stillwater, Oklahoma, during September through October of 2022, and housed them in clear plastic containers (height: 7.4 cm, diameter: 16.2 cm) in the laboratory. We housed spiders at a constant 25°C ± 1°C and 14L: 10D light regime and provided water *ad libitum*.

### 2.2 Prey Species

We purchased house crickets ( *Acheta domesticus* ) from a commercial distributor (Armstrong's Cricket Farm, Georgia USA). We housed crickets at a 14L: 10D light regime, provided water *ad libitum*, and maintained them on a dog food diet (Rachael Ray Nutrish Dog Food). This prey species was chosen because: (1) crickets are considered natural prey for wolf spiders, and (2) using prey that was reared on a known diet in controlled laboratory conditions may reduce intraspecific variation in prey nutrient content (Herzog et al. 2023).

### 2.3 Feeding Trials

To standardize hunger levels before feeding trials, all spiders were provided with 0.45–0.5 g of crickets and then fasted for 7 days. After this starvation period, each spider was offered a pre‐weighed cricket (or multiple crickets to reach the weight range) ranging from 0.3–0.35 g (Herzog et al. 2023). Spiders were allowed to feed for 24 h, after which any remaining live prey was removed. Each spider was used for multiple feeding trials, consisting of standardized feeding, a seven‐day fasting period, and a subsequent feeding trial. In total, seven feeding trials were conducted.

### 2.4 Spider Excretion Sample Collection

Spider excreta was collected 48 h after providing crickets, ensuring most excreta had been deposited (Barnes 2010; Herzog et al. 2023). We dissolved excreta in 1.5–2 mL of distilled water and transferred the solution into 2 mL centrifuge vials. The vials were centrifuged at 13,000 RPM for 10 min to form a pellet, then placed in an oven at 60°C for 48 h to fully evaporate the water. No liquid was removed prior to drying to ensure that we retained any dissolved nutrients. The dried excreta samples were then stored for further analysis.

### 2.5 Whole‐Body Sample Collection

For whole‐body elemental analysis, we collected adult male and female Carolina wolf spiders (*n* = 10 per sex) from fields in Stillwater, Oklahoma, in October 2023. Females that appeared visibly gravid (i.e., with noticeably swollen abdomens) were not collected. After capture, spiders were immediately frozen, and wet mass, carapace width, and leg length were recorded. Spiders were then dried at 60°C for 72 h before recording dry mass. Samples were then homogenized using a ball mill, and dried whole‐body samples were stored for further analysis. Although the specific diets of spiders prior to capture were unknown, the elemental composition of field‐collected spiders reflects the nutrient content that predators would consume and what would be released into the environment if spiders died naturally, making these measurements ecologically relevant.

### 2.6 Elemental Content Analysis of Spider Excretion and Whole Body

We measured the elemental content of male and female spider excreta (*n* = 23 for males, *n* = 35 for females) and whole bodies (*n* = 10 per sex). The samples were ground, homogenized, and subsampled for analysis. When excreta samples weighed less than 3 mg, they were randomly pooled with other low‐mass samples from different spiders of the same sex and trial to ensure sufficient material for each analysis, resulting in 20 pooled samples for males and 21 pooled samples for females. To evaluate whether pooling influenced variability or multivariate structure, we compared pooled and unpooled samples using several approaches. PERMDISP analyses showed no difference in dispersion for males (*p* = 0.526) and a small difference for females (*p* = 0.031). ANOVAs on PC1 and PC2 scores indicated that pooling had no effect on multivariate position (PC1: *p* = 0.581; PC2: *p* = 0.896). A PERMANOVA further showed that pooling did not significantly influence overall elemental composition (*p* = 0.075), and there was no Sex × Type interaction (*p* = 0.229). Together, these analyses demonstrate that pooling may have a slight effect on inter‐individual variability in female samples but did not affect the mean responses of males and females.

Subsamples were sent to the Cornell Stable Isotope Laboratory for carbon and nitrogen (C and N) measurements. Other elements (Ba, Ca, Cu, Fe, K, Li, Mg, Mn, Na, Ni, P, S, Si, Sr., and Zn) were analyzed using inductively coupled plasma–optical emission spectrometry (ICP‐OES; Thermo Scientific iCAP 7400) in the Department of Integrative Biology at Oklahoma State University. The elements included in our analyses represent all elements that could be reliably detected and quantified using our analytical methods. Trace elements remain understudied in spiders and other arthropods, so we intentionally took a broad, exploratory approach to capture the full measurable elemental profile. ICP‐OES sample preparation followed methods from Prater et al. (2020).

Elemental values that exceeded the upper detection limit (i.e., returned as NA) were replaced with the next highest observed value for that element. This is a conservative approach; hence we may be underestimating the concentration of some elements in products (e.g., Ca). When an element was measured on multiple wavelengths, any wavelength that returned exclusively negative values (i.e., all negative intensities) was dropped from the analysis, while remaining valid wavelengths for that element were retained. Due to problems with C measurements for male spiders at the Cornell Stable Isotope Laboratory, C was removed from further analysis due to low sample size (*n* = 4). However, N measurements were fine and retained for analysis for both sexes. Furthermore, a full mass‐balance analysis was not possible because spiders often deposited excreta on their legs and then groomed it off, preventing us from collecting complete samples.

### 2.7 Data Analysis

#### 2.7.1 Carolina Wolf Spider Body Measurements

We used Shapiro–Wilk tests to assess whether carapace width, leg length, dry mass, and wet mass of male and female wolf spiders were normally distributed (Table S1). Additionally, we used Levene's test to confirm homogeneity of variances across sexes for each trait (*p* > 0.05; Table S2). Based on these results, we conducted independent‐samples *t*‐tests to compare carapace width, leg length, dry mass, and wet mass between sexes (Table S3).

#### 2.7.2 Elemental Analysis

To evaluate multivariate differences in elemental balance across samples, we applied a centered log‐ratio (CLR) transformation to a compositional matrix containing all measured elements. This transformation preserves sub‐compositional coherence in data with a constant‐sum constraint (Aitchison 1986; Greenacre 2021). We then conducted a Principal Component Analysis (PCA) on the CLR‐transformed elemental composition of spider excreta and whole bodies to address collinearity among elements and visualize the influence of different products on elemental composition. Principal component values from the PCA were extracted for further statistical analysis, and ANOVAs were performed on the first and second principal components to assess the effect of sex on overall elemental composition in excreta and whole bodies. In addition, we conducted a permutational multivariate analysis of variance (PERMANOVA) on robust Aitchison distances of untransformed elemental data to evaluate the effects of sex, product, and their interaction.

Using the untransformed elemental concentrations (μg/g), we assessed normality using the Shapiro–Wilk test and variance homogeneity using Levene's test across sexes (male and female) and products (Tables S4–S7). For elements that met both assumptions, we used independent samples *t*‐tests to compare mean concentrations. When assumptions were violated, we applied Mann–Whitney *U* tests. These analyses evaluated the effects of sex and product on elemental concentrations (Table 4 and Tables S4–S7). Since differences between excreta and whole bodies were consistently observed, post hoc tests were not performed; instead, our primary focus was on elemental differences between sexes within each product.

## 3 Results

### 3.1 Male and Female Carolina Wolf Spider Comparison

T‐tests revealed significant differences between male and female wolf spiders in carapace width, wet mass, and dry mass (*p* < 0.05; Figure 1 and Table S3). On average, females were 42.3% heavier in wet mass, 37.1% heavier in dry mass, and had carapace widths 8.5% larger than males. Males had leg lengths 5.5% longer than females; however, this difference was not statistically significant (*p* > 0.05; Figure 1 and Table S3).

### 3.2 Principal Component Analysis

**FIGURE 2:**
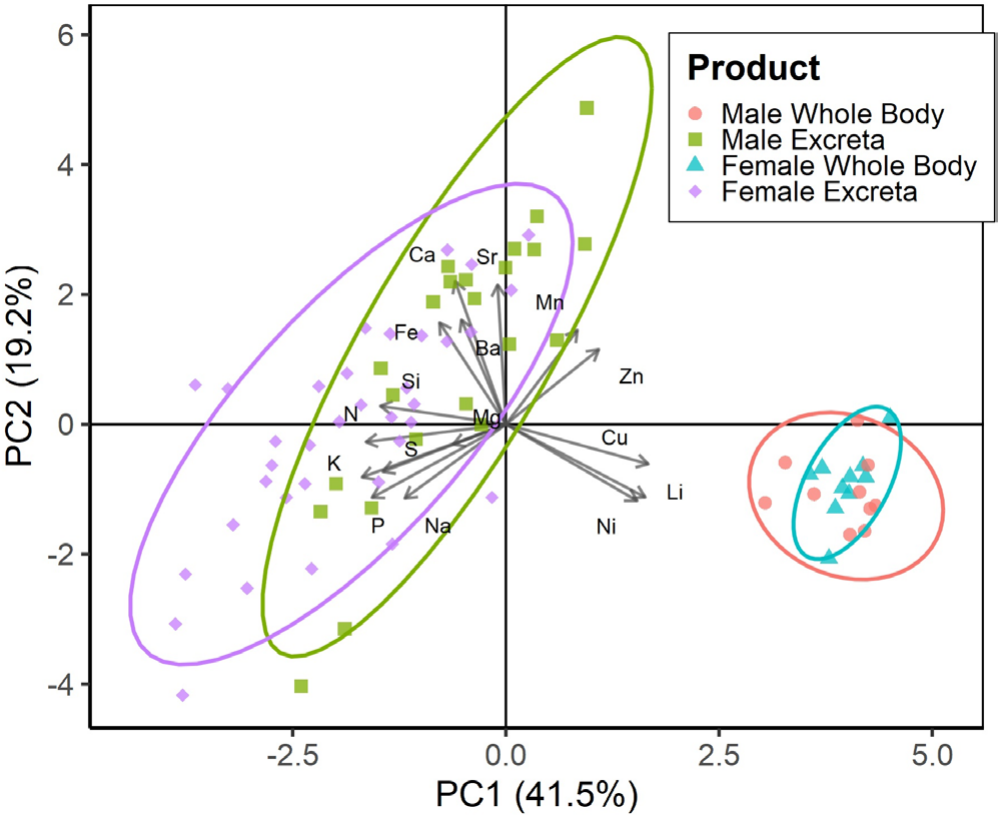
Principal component analysis (PCA) of the concentrations of 16 elements in *Hogna carolinensis* male and female whole bodies and excreta. The first two principal components are plotted on the x‐ and y‐axes. Eigenvectors indicate the direction and magnitude of concentration differences between groups. Large points represent group centroids, small points represent observed data, and ellipses denote 95% confidence intervals.

In the PCA conducted on the matrix of 16 CLR‐transformed elemental concentrations, the first three individual principal components accounted for 41.48% (PC1), 19.21% (PC2), and 8.56% (PC3) of the variance, respectively (Figure 2). We plotted elemental composition in PC1 × PC2 space, since these components captured the largest proportion of variance in two dimensions. On PC1, many elements had strong negative loadings (< −0.20), including Cu, Li, Mn, and Ni, while Ba, K, N, P, S, and Si had strong positive loadings (> 0.20) (Table 1). On PC2, several elements showed strong positive loadings (> 0.20), such as Na, P, and S, while others had strong negative loadings (< −0.20), including Ba, Ca, Mn, and Sr. (Table 1).

**TABLE 1:** Loading scores of elements for the first two principal components (PC1 and PC2) shown in Figure 2.

Element	PC1	PC2
Ba	0.249	−0.354
Ca	0.081	−0.254
Cu	−0.197	−0.169
Fe	0.131	−0.183
K	0.320	0.183
Li	−0.372	0.011
Mg	0.135	−0.099
Mn	−0.226	−0.231
N	0.367	−0.063
Na	0.151	0.269
Ni	−0.366	0.099
P	0.198	0.445
S	0.243	0.357
Si	0.339	−0.115
Sr	0.190	−0.441
Zn	−0.155	0.177

We visualized elemental composition by sex and product (i.e., excreta vs. whole body) in principal component space and observed clear separation between whole body and excreta samples (Figure 2). Excreta also exhibited greater variability between males and females compared to whole‐body samples (Figure 2). ANOVA on PC1 and PC2 scores revealed significant effects of product, sex, and their interaction on PC1 (*p* < 0.05), while only product and sex were significant for PC2 (*p* < 0.05; Table 2). Additionally, a PERMANOVA conducted on robust Aitchison distances of untransformed elemental data showed significant effects of product and sex (*p* < 0.005), but not their interaction (*p* > 0.05; Table 3).

**TABLE 2:** The results of ANOVA analysis on (A) PC1 and (B) PC2 axis values generated by principal components analysis.

	Df	Sum Sq	Mean Sq	Statistic	*p*
(A) PC1					
Product	1	421	421	452	* < 0.001
Sex	1	15.0	15.0	16.0	* < 0.001
Product:Sex	1	6.10	6.10	6.50	0.01
Residual	103	47.5	0.50		
(B) PC2					
Product	1	25.2	25.2	9.55	*0.003
Sex	1	10.7	10.7	4.04	0.05
Product:Sex	1	5.35	5.35	2.03	0.16
Residual	74	195	2.64		

*Note:* Significant *p*‐values are indicated with asterisks (*p < 0.05).

**TABLE 3:** PERMANOVA results on the effect of product, sex, and product by sex.

	Df	Sum Sq	*R* Sq	Statistic	*p*
Product	1	320	0.49	74.4	*0.001
Sex	1	28.8	0.04	6.68	*0.001
Product:Sex	1	6.75	0.01	1.57	0.18
Residual	68	293	0.45		
Total	71	648	1		

*Note:* Significant *p*‐values are indicated with asterisks (*p < 0.05).

### 3.3 Individual Element Comparison

Analyses using independent‐samples *t*‐tests and Mann–Whitney *U* tests identified significant differences between males and females in excreta concentrations of Ca, K, Mn, P, Si, and Zn, and in whole‐body concentrations of Fe, Ni, S, and Sr. (*p* < 0.05; Table 4). Additionally, irrespective of sex, product type strongly influenced elemental composition. Wolf spider excreta contained higher concentrations of Ba, Ca, Fe, K, N, Na, P, S, and Si relative to whole bodies, while whole bodies contained higher concentrations of Cu, Li, and Ni (Figure 2 and Tables 2, 3, 4).

**TABLE 4:** Mean (±SE) elemental concentrations (μg/mg) in whole‐body tissue and excreta of male and female *Hogna carolinensis* . Percent difference (% Diff M–F) reflects the relative difference between male and female means.

	Mean (M)	SE (M)	Mean (F)	SE (F)	% Diff M–F	Test	Df1	Statistic	*p*
*Whole body*									
Ba	0.004	0.001	0.003	0.0005	33.3	MW‐U	NA	59	0.529
Ca	0.618	0.039	0.588	0.049	5.1	T	18	−0.473	0.642
Cu	0.074	0.009	0.068	0.008	8.82	T	18	−0.515	0.613
Fe	0.068	0.015	0.032	0.003	113	MW‐U	NA	8	0.0007**
K	5.88	0.204	5.55	0.269	5.95	T	18	−0.961	0.349
Li	0.003	0.0005	0.004	0.0005	−25	T	18	1.1	0.286
Mg	0.781	0.041	0.826	0.049	−5.45	T	18	0.711	0.486
Mn	0.013	0.001	0.011	0.001	18.2	T	18	−1.42	0.172
N	124	2.9	122	2.7	1.64	MW‐U	NA	44	0.677
Na	3	0.126	3.01	0.163	−0.332	T	18	0.049	0.962
Ni	0.016	0.004	0.009	0.0006	77.8	MW‐U	NA	17	0.011*
P	7.05	0.255	6.81	0.287	3.52	T	18	−0.617	0.545
S	6.04	0.174	5.37	0.288	12.5	MW‐U	NA	18	0.015*
Si	0.021	0.002	0.02	0.004	5	T	18	−0.386	0.704
Sr	0.003	0.0002	0.004	0.0004	−25	MW‐U	NA	86	0.005**
Zn	0.221	0.011	0.233	0.023	−5.15	MW‐U	NA	46	0.796
*Excreta*									
Ba	0.007	0.0008	0.007	0.0009	0	MW‐U	NA	377	0.693
Ca	1.76	0.212	1.29	0.169	36.4	MW‐U	NA	263	0.026*
Cu	0.025	0.002	0.02	0.002	25	MW‐U	NA	296	0.092
Fe	0.131	0.019	0.133	0.015	−1.5	MW‐U	NA	405	0.975
K	25.1	2.98	34	2.23	−26.2	T	56	2.42	0.019*
Li	0.00026	0.00002	0.0003	0.00003	4	MW‐U	NA	307	0.132
Mg	1.19	0.079	1.17	0.111	1.71	MW‐U	NA	341	0.335
Mn	0.026	0.003	0.012	0.002	117	MW‐U	NA	166	0.0001***
N	315	7.43	324	7.23	−2.78	T	56	0.847	0.401
Na	10.7	1.46	10.4	0.977	2.88	T	56	−0.181	0.857
Ni	0.0005	0.00006	0.001	0.0004	−45.4	MW‐U	NA	427	0.705
P	15.8	1.29	19.2	0.961	−17.7	T	56	2.13	0.037*
S	11.8	1.07	15.2	1.22	−22.4	MW‐U	NA	506	0.102
Si	0.163	0.023	0.093	0.013	75.3	MW‐U	NA	196	0.0008**
Sr	0.006	0.0008	0.006	0.0009	0	MW‐U	NA	350	0.412
Zn	0.394	0.086	0.155	0.034	154	MW‐U	NA	201	0.001**

*Note:* Statistical differences were assessed using two‐sample *t*‐tests (T) or Mann–Whitney *U* tests (MW). Significant *p*‐values are indicated with bolded text and the level of significance with asterisks (*p < 0.05, **p < 0.01, ***p < 0.001).

## 4 Discussion

We hypothesized that male and female Carolina wolf spiders would exhibit significant differences in elemental concentrations in both whole‐body and excreta samples, potentially reflecting intraspecific variation and differing life history strategies. This hypothesis was only partially supported. While sex and product significantly influenced the concentrations of some elements, the multivariate separation between male and female whole bodies was minimal, indicating limited overall variation (Figure 2 and Tables 2, 3, 4). Despite this modest separation, clear sex‐specific differences emerged at the level of individual elements. Specifically, male wolf spiders excreted higher concentrations of Ca, Mn, Si, and Zn, while females excreted higher concentrations of K and P. In whole‐body samples, males had higher concentrations of Fe, Ni, and S, whereas females had higher concentrations of Sr. (Table 4). Regardless of sex, both males and females excreted higher concentrations of Ba, Ca, Fe, K, N, Na, P, S, Si relative to whole bodies, which is likely because whole bodies of animals have higher concentrations of C that dilutes micronutrients relative to excreta. Conversely, both sexes retained Cu, Li, and Ni in their whole bodies and had lower concentrations of these elements in their excreta, consistent with previous research (Figure 2) (Herzog et al. 2023). Our findings highlight the complexity of intraspecific variation in elemental composition and emphasize the need for further research into how such variation influences consumer‐driven nutrient cycling and broader ecosystem functioning.

In partial support of our initial hypothesis, whole‐body elemental content significantly varied for four elements between male and female Carolina wolf spiders (i.e., Fe, Ni, S, and Sr) (Table 4). While studies on sex‐based differences in elemental content among invertebrates remain limited, previous research has identified elemental differences between sexes for some species (Goos et al. 2017; Sobcyzk et al. 2020; Filipiak et al. 2021). For spiders specifically, Sobcyzk et al. (2020) found that both sex and family may influence a spider's stoichiometric phenotype. It is plausible that increasing sexual dimorphism within a species corresponds to greater differences in whole‐body elemental content between sexes. Compared to species in other spider families (i.e., Araneidae, Nephilidae, Theridiidae, etc.) wolf spiders exhibit a lower degree of sexual dimorphism (Foellmer and Moya‐Larano 2007; Cordellier et al. 2020; Kuntner and Coddington 2020) (Figure 1 and Table S3). This reduced dimorphism may explain some absences of sex‐based differences in whole‐body elemental content observed in this study. Given the breadth of sexual dimorphism that exists throughout spider phylogeny, future research should focus on quantifying sexual dimorphism to assess whether its degree predicts stoichiometric differences between sexes. Such findings could be particularly relevant for understanding how shifts in sex ratios or loss of a species may affect consumer‐driven nutrient cycles.

The physiological and ecological mechanisms driving the retention of specific elements observed in this study, such as Cu, Fe, Li, Ni, and Sr., are largely unclear and require further study (Figure 2). For example, Sr. can substitute for Ca in mineralized tissues and has been detected in the exoskeletons of some arthropods (e.g., Brannon and Rao 1979), suggesting a possible structural role that may contribute to the higher whole‐body Sr. concentrations observed in females, especially given their larger body size relative to males (Figure 1). In contrast, Cu‐containing proteins aid in oxygen transport within the hemolymph of spiders, which may explain the retained high Cu concentrations in whole bodies (Foelix 1996; Nentwig 2012; Rehm et al. 2012). In contrast, the physiological roles of Li and Ni remain unclear. Previous studies suggest that wolf spiders can bioaccumulate certain metals (e.g., Cd, and Pb.) which can have deleterious fitness effects at higher concentrations (Hendrickx et al. 2003; Jung and Lee 2012; Butt and Aziz 2016; Stojanowska et al. 2020). Thus, the observed whole‐body Li and Ni concentrations may be a result of bioaccumulation of pollutants present in *H. carolinensis* habitats. Furthermore, the higher concentrations of Ni (and possibly Fe) detected in males may result from increased movement across contaminated substrates during mate searching and subsequent leg or pedipalp cleaning, leading to ingestion of heavy metals (Table 4) (Foelix 2011; Trabalon 2022). However, the ecological consequences of such bioaccumulation, particularly its effects on trophic cascades, warrant further investigation, especially for organisms that prey on wolf spiders (i.e., other spiders, anurans, birds, etc.) (Richman and Jackson 1992; Gunnarsson 2007; Nyffeler and Pusey 2014).

There were several elements excreted at higher concentrations for both sexes (i.e., Ba, Ca, Fe, K, N, Na, P, S, and Si), and significant differences between male and female wolf spiders for Ca, K, Mn, P, Si, and Zn in excreta elemental concentrations (Figure 2 and Table 4). Notably, male wolf spiders excreted higher concentrations of Ca, Mn, Si, and Zn whereas females excreted higher concentrations of K and P. The importance of N, P, and K in consumer‐driven nutrient cycles and their effects across trophic levels is well documented (Andersson et al. 1988; Hilderbrand et al. 1999; Sterner and Elser 2002; Vanni 2002; Vanni et al. 2002; Schmitz et al. 2010; Elser et al. 2016; Kazama 2019). Female wolf spiders may be contributing higher concentrations of N, P, and K to lower trophic levels via nutrient deposition. Additionally, extensive evidence demonstrates that some micronutrients (i.e., trace elements) can significantly alter community structure (Prather et al. 2018; Welti et al. 2019; Reihart et al. 2020; Kaspari 2021; Welti and Kaspari 2021). Given that spiders occupy nearly all terrestrial ecosystems and consume 400–800 million tons of prey annually (Nyffeler and Birkhofer 2017), their role in consumer‐driven nutrient cycles is substantial. Furthermore, there is a growing body of literature demonstrating the role of spiders in nutrient dynamics and their effects on overall ecosystem function (Hodkinson et al. 2001; Romero et al. 2006; Hawlena et al. 2012; Buchkowski and Schmitz 2015; Ludwig et al. 2018; Trubl and Johnson 2019; Sagi and Hawlena 2021; Herzog et al. 2023; Wilder et al. 2025). Thus, further investigation into spider‐mediated micronutrient cycling and its effects on terrestrial food webs is needed, particularly for trace elements such as Ca, Mn, Si and Zn, whose roles in physiological functions and food webs remain less well understood for invertebrates including spiders.

Our findings reveal some sex‐based differences in micronutrient excretion and whole‐body elemental concentrations among Carolina wolf spiders, highlighting the potential role of intraspecific differences in consumer‐driven nutrient cycles. Significant differences between whole‐body (i.e., Fe, Ni, S, and Sr) and excreta (i.e., Ca, K, Mn, P, Si, and Zn) elemental concentrations suggest that male and female wolf spiders may contribute differently to micronutrient flows within ecosystems. These results emphasize the need for further research into how sexual dimorphism and life history strategies influence stoichiometric phenotypes across taxa, as well as potential nutrient feedback loops associated with these variables. In some species of invertebrates, adult females are present in the population for a longer period than adult males (Criscuolo et al. 2010; Sielezniew et al. 2020). Hence, future studies should explore the ecological consequences of intraspecific differences, including seasonal and trophic‐level impacts, to better understand consumer‐driven micronutrient cycling in food webs. By integrating predator stoichiometry with ecological nutrient dynamics, we can refine our understanding of consumer‐driven cycles and the potential consequences of demographic shifts on ecosystem functioning. Additionally, the roles of understudied elements deposited by wolf spiders, such as Ca, Mn, Si and Zn, warrant further investigation to fully assess their ecological significance.

## Author Contributions

**Colton Herzog:** conceptualization (equal), data curation (equal), formal analysis (equal), investigation (equal), methodology (equal), validation (equal), visualization (equal), writing – original draft (equal), writing – review and editing (equal). **Jamie T. Reeves:** conceptualization (equal), data curation (equal), formal analysis (equal), methodology (equal), validation (equal), visualization (equal), writing – original draft (equal), writing – review and editing (equal). **Shawn M. Wilder:** conceptualization (equal), data curation (equal), formal analysis (equal), funding acquisition (lead), investigation (equal), methodology (equal), resources (equal), validation (equal), visualization (equal), writing – original draft (equal), writing – review and editing (equal).

## Funding

Funding was obtained through NSF IOS–2420366.

## Conflicts of Interest

The authors declare no conflicts of interest.

## Supporting information


**Table S1:** Results of Shapiro–Wilk tests assessing normality for carapace width, leg length, dry mass, and wet mass.
**Table S2:** Results of Levene's tests assessing homogeneity of variances between sexes for carapace width, leg length, dry mass, and wet mass.
**Table S3:** Results of *t*‐tests comparing male and female wolf spiders for carapace width, leg length, dry mass, and wet mass.
**Table S4:** Test statistics for Shapiro–Wilk's test for normality showing the effect of sex on the distribution of excreta elemental content.
**Table S5:** Test statistics for Shapiro–Wilk's test for normality showing the effect of sex on the distribution of whole‐body elemental content.
**Table S6:** Test statistics for Levene's test results for homogeneity of variances showing the effects of sex on the variance of excreta individual elemental content.
**Table S7:** Test statistics for Levene's test results for homogeneity of variances showing the effect of sex on the variance of whole‐body elemental content.

## Data Availability

The data and R code supporting the findings of this study are provided at https://doi.org/10.6084/m9.figshare.30680567.
