# Supplementary material for: Testing for Differences in Consumer‐Based Nutrient Cycling Between Male and Female Wolf Spiders ( Hogna carolinensis )
Source: Ecol Evol. 2025 Dec 5;15(12):e72640. doi: 10.1002/ece3.72640 (PMC12679667; doi:10.1002/ece3.72640)
Supplement: Supplementary file 1 — Table S1: Results of Shapiro–Wilk tests assessing normality for carapace width, leg length, dry mass, and wet mass. Table S2: Results of Levene's tests assessing homogeneity of variances between sexes for carapace width, leg length, dry mass, and wet mass. Table S3: Results of t‐tests comparing male and female wolf spiders for carapace width, leg length, dry mass, and wet mass. Table S4: Test statistics for Shapiro–Wilk's test for normality showing the effect of sex on the distribution of excreta elemental content. Table S5: Test statistics for Shapiro–Wilk's test for normality showing the effect of sex on the distribution of whole‐body elemental content. Table S6: Test statistics for Levene's test results for homogeneity of variances showing the effects of sex on the variance of excreta individual elemental content. Table S7: Test statistics for Levene's test results for homogeneity of variances showing the effect of sex on the variance of whole‐body elemental content. [file ECE3-15-e72640-s001.docx]

| Variable | Sex | W | P |
| --- | --- | --- | --- |
| Carapace width | Female | 0.893 | 0.181 |
| Carapace width | Male | 0.939 | 0.546 |
| Leg length | Female | 0.928 | 0.429 |
| Leg length | Male | 0.947 | 0.637 |
| Dry mass | Female | 0.895 | 0.191 |
| Dry mass | Male | 0.930 | 0.445 |
| Wet mass | Female | 0.927 | 0.414 |
| Wet mass | Male | 0.918 | 0.338 |

Table S1: Results of Shapiro–Wilk tests assessing normality for carapace width, leg length, dry mass, and wet mass.

| Variable | F | P |
| --- | --- | --- |
| Carapace width | 2.068 | 0.168 |
| Leg length | 0.443 | 0.514 |
| Dry mass | 0.073 | 0.791 |
| Wet mass | 0.946 | 0.344 |

Table S2: Results of Levene’s tests assessing homogeneity of variances between sexes for carapace width, leg length, dry mass, and wet mass.

| Variable | T Stat | df | P |
| --- | --- | --- | --- |
| Carapace width | 2.27 | 18 | 0.036 |
| Leg length | -1.33 | 18 | 0.199 |
| Dry mass | 3.35 | 18 | 0.004 |
| Wet mass | 4.16 | 18 | 0.0006 |

Table S3: Results of t-tests comparing male and female wolf spiders for carapace width, leg length, dry mass, and wet mass.

|  | Variable | W | P |
| --- | --- | --- | --- |
| 1 | Ba | 0.89 | .0001 |
| 2 | Ca | 0.87 | <0.001 |
| 3 | Cu | 0.92 | 0.001 |
| 4 | Fe | 0.93 | 0.002 |
| 5 | K | 0.99 | 0.747 |
| 6 | Li | 0.88 | <0.001 |
| 7 | Mg | 0.93 | 0.004 |
| 8 | Mn | 0.87 | <0.001 |
| 9 | N | 0.97 | 0.096 |
| 10 | Na | 0.96 | 0.078 |
| 11 | Ni | 0.28 | <0.001 |
| 12 | P | 0.98 | 0.324 |
| 13 | S | 0.94 | 0.009 |
| 14 | Si | 0.80 | <0.001 |
| 15 | Sr | 0.80 | <0.001 |
| 16 | Zn | 0.78 | <0.001 |

Table S4: Test statistics for Shapiro-Wilk’s test for normality showing the effect of sex on the distribution of excreta elemental content.

|  | Variable | W | P |
| --- | --- | --- | --- |
| 1 | Ba | 0.79 | 0.001 |
| 2 | Ca | 0.94 | 0.234 |
| 3 | Cu | 0.95 | 0.408 |
| 4 | Fe | 0.62 | <0.001 |
| 5 | K | 0.94 | 0.266 |
| 6 | Li | 0.95 | 0.349 |
| 7 | Mg | 0.96 | 0.635 |
| 8 | Mn | 0.91 | 0.061 |
| 9 | N | 0.88 | 0.015 |
| 10 | Na | 0.94 | 0.287 |
| 11 | Ni | 0.57 | <0.001 |
| 12 | P | 0.96 | 0.568 |
| 13 | S | 0.88 | 0.021 |
| 14 | Si | 0.91 | 0.051 |
| 15 | Sr | 0.90 | 0.036 |
| 16 | Zn | 0.89 | 0.031 |

Table S5: Test statistics for Shapiro-Wilk’s test for normality showing the effect of sex on the distribution of whole-body elemental content.

|  | Variable | Levene Stat | P |
| --- | --- | --- | --- |
| 1 | Ba | 0.59 | 0.444 |
| 2 | Ca | 0.33 | 0.567 |
| 3 | Cu | 0.56 | 0.457 |
| 4 | Fe | 0.06 | 0.816 |
| 5 | K | 0.03 | 0.860 |
| 6 | Li | 2.73 | 0.104 |
| 7 | Mg | 3.07 | 0.085 |
| 8 | Mn | 1.05 | 0.309 |
| 9 | N | 0.27 | 0.608 |
| 10 | Na | 2.18 | 0.150 |
| 11 | Ni | 0.78 | 0.380 |
| 12 | P | 0.03 | 0.867 |
| 13 | S | 1.10 | 0.298 |
| 14 | Si | 3.81 | 0.056 |
| 15 | Sr | 0.33 | 0.566 |
| 16 | Zn | 6.33 | 0.015 |

Table S6: Test statistics for Levene’s test results for homogeneity of variances showing the effects of sex on the variance of excreta individual elemental content.

|  | Variable | Levene Stat | P |
| --- | --- | --- | --- |
| 1 | Ba | 0.91 | 0.354 |
| 2 | Ca | 0.12 | 0.728 |
| 3 | Cu | 0.20 | 0.660 |
| 4 | Fe | 1.93 | 0.181 |
| 5 | K | 0.06 | 0.813 |
| 6 | Li | <0.001 | 1.000 |
| 7 | Mg | 0.42 | 0.525 |
| 8 | Mn | 0.002 | 0.965 |
| 9 | N | 0.19 | 0.668 |
| 10 | Na | 1.32 | 0.266 |
| 11 | Ni | 1.29 | 0.270 |
| 12 | P | 0.09 | 0.764 |
| 13 | S | 0.60 | 0.450 |
| 14 | Si | 1.16 | 0.296 |
| 15 | Sr | 0.24 | 0.631 |
| 16 | Zn | 1.20 | 0.288 |

Table S7: Test statistics for Levene’s test results for homogeneity of variances showing the effect of sex on the variance of whole-body elemental content.
